# Production of scFv-Conjugated Affinity Silk Powder by Transgenic Silkworm Technology

**DOI:** 10.1371/journal.pone.0034632

**Published:** 2012-04-04

**Authors:** Mitsuru Sato, Katsura Kojima, Chisato Sakuma, Maria Murakami, Eriko Aratani, Takato Takenouchi, Yasushi Tamada, Hiroshi Kitani

**Affiliations:** 1 Animal Immune and Cell Biology Research Unit, National Institute of Agrobiological Sciences, Tsukuba, Ibaraki, Japan; 2 Silk Materials Research Unit, National Institute of Agrobiological Sciences, Tsukuba, Ibaraki, Japan; Technical University of Braunschweig, Germany

## Abstract

*Bombyx mori* (silkworm) silk proteins are being utilized as unique biomaterials for medical applications. Chemical modification or post-conjugation of bioactive ligands expand the applicability of silk proteins; however, the processes are elaborate and costly. In this study, we used transgenic silkworm technology to develop single-chain variable fragment (scFv)-conjugated silk fibroin. The cocoons of the transgenic silkworm contain fibroin L-chain linked with scFv as a fusion protein. After dissolving the cocoons in lithium bromide, the silk solution was dialyzed, concentrated, freeze-dried, and crushed into powder. Immunoprecipitation analyses demonstrate that the scFv domain retains its specific binding activity to the target molecule after multiple processing steps. These results strongly suggest the promise of scFv-conjugated silk fibroin as an alternative affinity reagent, which can be manufactured using transgenic silkworm technology at lower cost than traditional affinity carriers.

## Introduction

*Bombyx mori* (silkworm) silk has been recognized as a unique natural biopolymer for various biomedical applications. After silk fibroin fibers are dissolved in aqueous solution, this protein can be fabricated into various material formats, such as powder, fibers, gels, sponges, or thin films [1], [2], [3], [4]. In addition to using the natural fibroin protein, this protein can be chemically modified [5], [6], [7] or post-conjugated with bioactive ligands [8], [9], [10] to alter its physical or biological properties. For instance, the coupling of an RGD sequence has been demonstrated to enhance cell adhesion to the silk fibroin film [8], [9], and bone morphogenetic protein-2 (BMP-2)-decorated silk fibroin films induce osteogenic differentiation of human bone marrow stromal cells [10]. However, the modification procedure is often accompanied by technical difficulties, and high manufacturing costs are inevitable.

Recent advances in transgenic silkworm technology have demonstrated that recombinant proteins can be produced in the silk glands, either independently from the silk proteins [11], [12], or fused with fibroin proteins [13], [14], [15]. The latter strategy was applied in the transgenic silkworm, which produces silk containing enhanced green fluorescent protein (EGFP) [13], [15] and basic fibroblast growth factor (bFGF) [14]. These results suggest that the recombinant protein is able to retain its original structure and function even when fused to silk fibroin proteins. To expand the applicability of transgenic silk fibroins as a novel affinity reagent, we sought to generate a transgenic silkworm that spins antibody-conjugated silk fibroins. However, the intact antibody is a large, multiplex protein composed of immunoglobulin H- and L-chains interlinked with disulfide bonds. Due to the size and complexity of the antibody, the design of a single fusion protein composed of whole antibody molecule and fibroin proteins is unlikely. In addition, the isolation and purification of silk fibroins generally require multiple steps, including degumming, solubilization, and dialysis, and these treatments would irreversibly destroy the antibody's biological activity.

However, advances in genetic engineering technology have demonstrated that the antibody can be dissected and reformatted into smaller units, such as Fab, scFv, or single-domain antibody [16], [17], [18], [19]. Of these smaller antibody formats, the single-chain variable fragment (scFv), which is composed of V_H_ and V_L_ domains, has several biophysical advantages over the original antibody format. For example, some but not all of scFv are able to retain its specific binding activity when it is expressed in the cytoplasm [20], suggesting that the proper conformation of the V_H_ and V_L_ domains are well maintained in strongly reducing conditions. Therefore, the scFv antibody format may be suitable not only because of its compactness, but also because of its tolerance to engineering (such as conjugation to other proteins, followed by multi-step physical and chemical processing).

In this study, we generated a transgenic silkworm strain that produces silk fibroin protein fused to scFv. The scFv construct was derived from a monoclonal antibody (mAb) against Wiskott-Aldrich syndrome protein (WASP), which is an important immune adaptor molecule in mammals [20], [21], [22], [23]. The present work demonstrates the promising possibility of scFv-conjugated silk fibroin proteins as a unique alternative to conventional affinity reagents.

## Results

### Transgenic silkworms produce genetically engineered fibroin protein in silk powder

**Figure 1:**
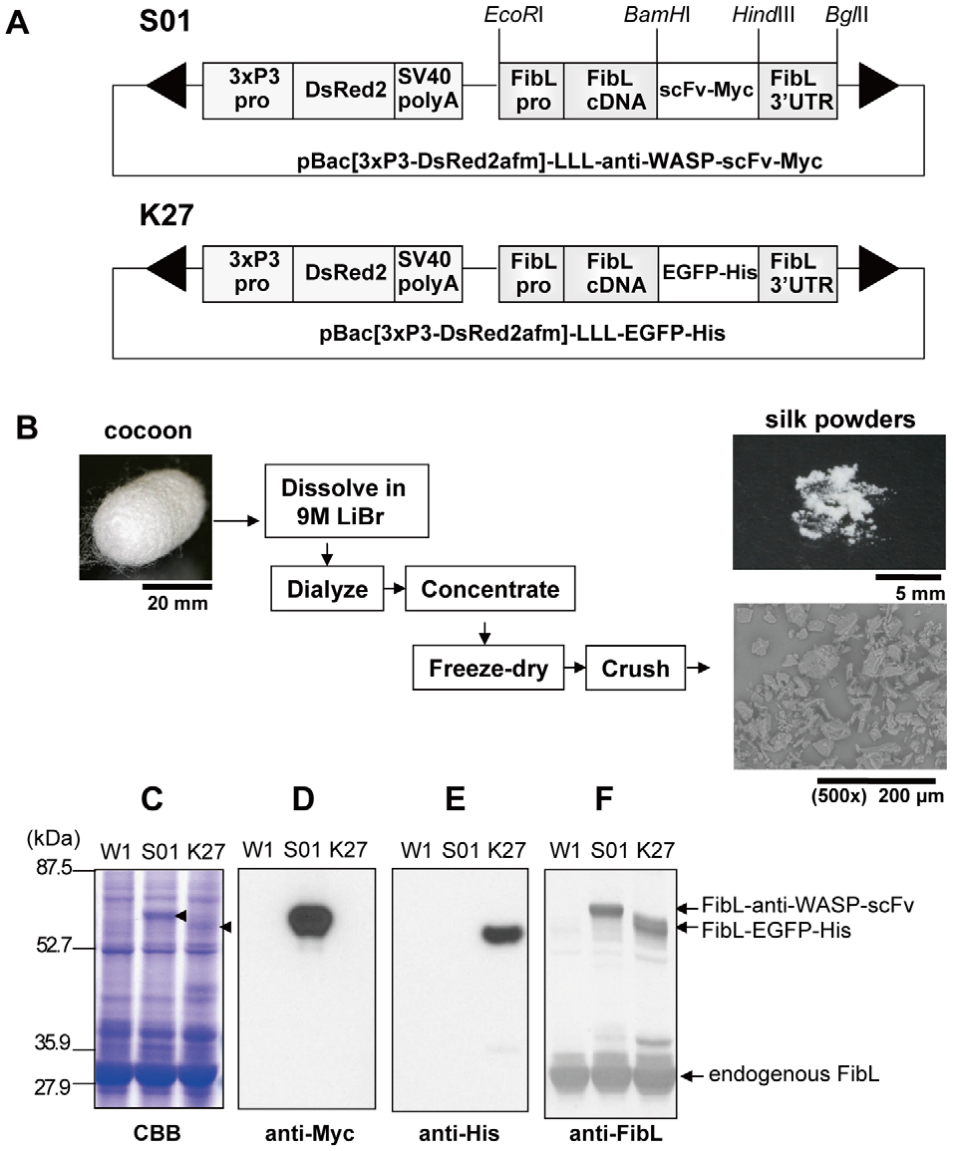
Construction of plasmid for transgenic silkworms and production of genetically engineered fibroin proteins in silk powder. (A) Schematic representation of the DNA plasmids for S01 and K27 transgenic silkworm strains. Each plasmid contains expression units for selection marker and recombinant proteins between the *piggyBac* repeated terminal sequences (arrowheads). Shown are the 3xP3 promoter (3xP3pro), DsRed2 gene, SV40 polyA signal sequence (SV40 polyA), fibrion L-chain promoter (FibLpro), cDNA of fibroin L-chain (FibL cDNA), cDNA of anti-WASP-scFv fused with a Myc-tag sequence (scFv-Myc), EGFP cDNA fused with 6×His (EGFP-His), and fibroin L-chain 3′-untranslated region (FibL-3′UTR). The restriction enzyme sites are indicated for *Eco*RI, *Bam*HI, *Hind*III, and *Bgl*II. (B) A schematic procedure for preparation of silk powder. Cocoons produced by silkworms were processed into silk powder. Silk powder from each silkworm strain was observed and photographed at 500× using low-vacuum scanning electron microscopy. (C, D, E, and F) SDS-PAGE and Western blot analysis showing expression of the transgenes FibL-anti-WASP-scFv-Myc (S01) and FibL-EGFP-His (K27) in silk powder. Silk powder derived from wild-type (W1), S01, and K27 strains was lysed and separated by SDS-PAGE, and followed by CBB staining (C). The arrowheads indicate the expression of FibL-anti-WASP-scFv-Myc (S01) and FibL-EGFP-His (K27). Immunoblots were probed with anti-Myc-tag pAb (D), anti-His pAb (E), and anti-FibL pAb (F).

We established two transgenic silkworm strains, S01 and K27, which spun silk containing fibroin L-chain conjugated with scFv and EGFP, respectively (Table 1 and Figure 1A). Cocoons produced by wild-type w1-pnd (W1), transgenic S01 and K27 silkworms were chopped, dissolved in LiBr solution, dialysed, freeze-dried, and fabricated into silk powder, as described in Materials and Methods. Powder derived from each silk strain showed similar morphology: amorphous fragments measuring 1–40 µm in diameter (Figure 1B). The composition of the silk powder is considered to be similar to that of silk fibers in cocoons; sericin (20% w/w), fibroin H-chain (72.2% w/w), fibroin L-chain (6.8% w/w), and fibrohexamerin(fhx)/P25 (1% w/w).

**Table 1:** Outcome of transgenesis.

Number					Percent
Strain	Injectedeggs	Hatchedlarvae^a^	G1 broods^b^	Broods with DsRed2- positive larvae^c^	Success rate of transgenesis
S01	794	655	162	7	4.3
K27	1443	440	47	11	23.4

a Hatched larvae (G0) were allowed to develop into moths.

b The moths were intercrossed, and the resulting G1 broods were counted.

c Embryos from G1 broods were screened for DsRed2 fluorescence.

The expression of the transgenes FibL-anti-WASP-scFv-Myc (S01) and FibL-EGFP-His (K27) in each silk powder were confirmed by sodium dodecyl sulfate polyacrylamide gel electrophoresis (SDS-PAGE), followed by Coomassie brilliant blue (CBB) staining (Figure 1C) and Western blotting with anti-Myc (Figure 1D), anti-His (Figure 1E), or anti-FibL (Figure 1F) antibodies. Semi-quantitative analysis using an anti-FibL polyclonal antibody (pAb) revealed that 25% of endogenous fibroin L-chain was substituted with the scFv- or EGFP-conjugated fibroin L-chain in the transgenic silk powders (Figure 1F). These results suggest that both scFv and EGFP constructs fused with fibroin L-chain are efficiently expressed as fusion proteins and integrated into silk fibers.

### Affinity purification of the target molecule by the specific transgenic silk powder

**Figure 2:**
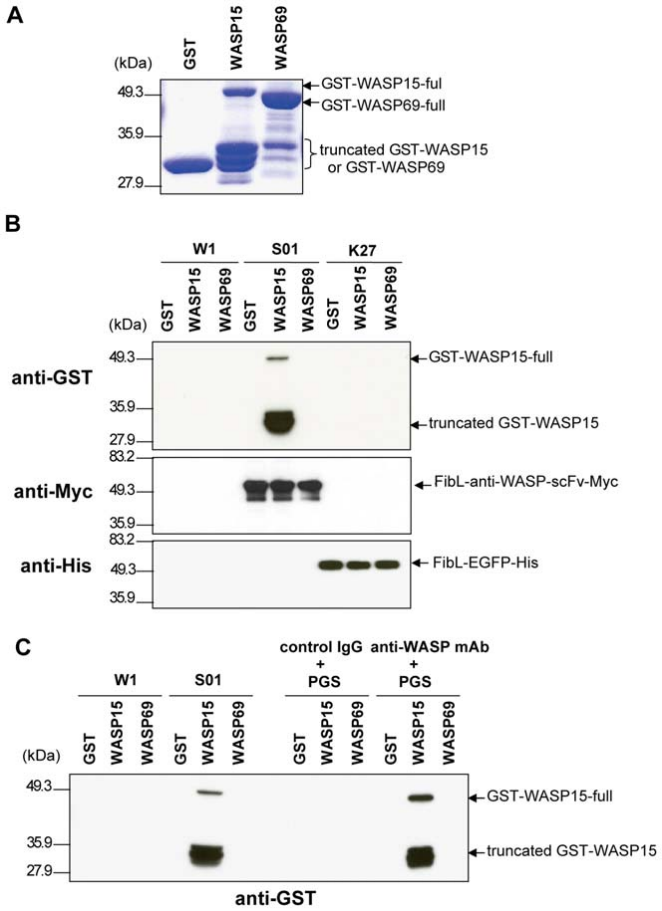
Affinity purification of the target molecule using transgenic silk powder. (A) The properties of the probe proteins GST, GST-WASP15 (WASP15), and GST-WASP69 (WASP69) were confirmed by SDS-PAGE followed by CBB staining. (B) The probe proteins were incubated and precipitated using silk powder derived from W1, S01, and K27 strains. Their specific binding was analyzed by immunoblotting with anti-GST antibody. The expression of FibL-anti-WASP-scFv-Myc in S01 and FibL-EGFP-His in K27 silk powder was confirmed by immunoblotting with anti-Myc pAb or anti-His pAb, respectively. (C) Silk powder (from W1 and S01 strains) or protein G-sepharose (PGS) coupled with anti-WASP mAb or control mouse IgG were incubated with the probe proteins GST, GST-WASP15, and GST-WASP69. Immune complexes were analyzed by Western blotting with anti-GST antibody. Immunoblots are representative of three independent experiments.

To test the specific affinity of scFv-conjugated silk powder to the target protein, we performed a series of *in vitro* binding assays using GST alone, or GST-WASP15 and GST-WASP69 fusion proteins produced and affinity purified from *E.coli* cells. The properties of these target proteins were confirmed by SDS-PAGE, followed by CBB staining. GST and the majority of GST-WASP69 were detected as full-size proteins (30 kDa and 48 kDa, respectively; Figure 2A). GST-WASP15 (52 kDa in full-size), the specific antigen against which the parental mAb of anti-WASP-scFv [20] was raised, was predominantly detected in several truncated forms (30–34 kDa), likely because of post-translational processing or degradation in *E. coli* cells (Figure 2A).

Binding assays demonstrated that the specific interaction was detected only in the incubation set of S01 silk powder and its target protein, GST-WASP15, and not in other protein constructs (Figure 2B, top panel). Using this binding assay, the equivalent expression level of FibL-anti-WASP-scFv-Myc and FibL-EGFP-His in each silk powder was confirmed by Western blotting with anti-Myc and anti-His antibodies, respectively (Figure 2B, middle and bottom panels). These results suggest that S01 silk powder exhibits specific binding activity to the target molecule, likely through the function of anti-WASP-scFv-conjugated fibroin L-chains, which are integrated into the S01 silk powder.

### Equivalent immunoprecipitation potency of scFv-conjugated fibroin and its parental mAb-coupled protein G-sepharose

To compare the binding ability of anti-WASP-scFv–conjugated silk powder to that of a conventional immunoprecipitation reagent, silk powders from W1 and S01 strains or protein G-sepharose coupled with anti-WASP mAb or control mouse IgG were incubated with purified probe proteins, GST, GST-WASP15, and GST-WASP69. The immunocomplexes were pulled down and analyzed by Western blotting with anti-GST antibody. In this assay, S01 silk powder particle immunoprecipitated its target probe, GST-WASP15, as efficiently as anti-WASP-mAb–coupled protein G-sepharose (Figure 2C); by contrast, W1 silk powder particle did not react to any of these probe proteins. These results strongly suggest that anti-WASP-scFv–conjugated fibroin L-chain in S01 silk powder has equivalent immunoprecipitation potency compared with its parental mAb-coupled protein G-sepharose.

### ScFv-conjugated silk powder particle efficiently immunoprecipitates native WASP in RAW264.7 cell lysate

**Figure 3:**
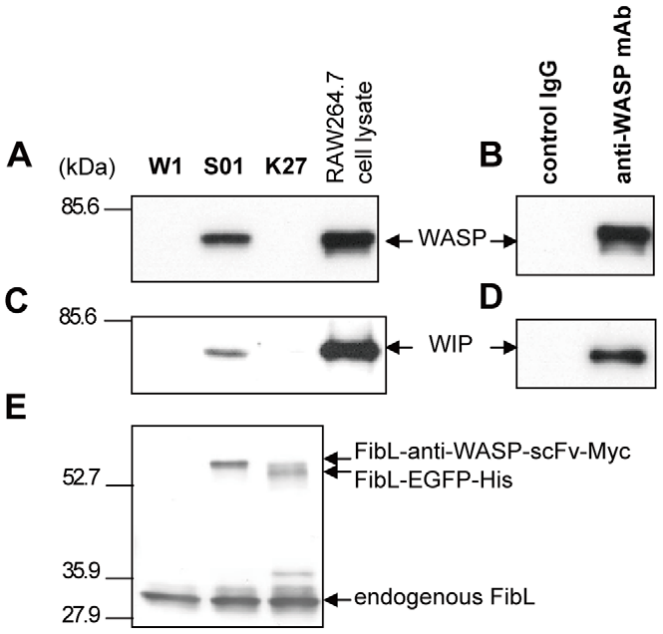
Transgenic silk powder particle immunoprecipitates native WASP from mouse macrophage extracts. A mouse macrophage RAW 264.7 cell line was lysed and incubated separately with silk powder particle from W1, S01, or K27 strains, or with protein G-sepharose (PGS) coupled with anti-WASP mAb or control mouse IgG. Immunocomplexes were analyzed by Western blotting with anti-WASP (A and B), and anti-WIP antibodies (C and D). The expression levels of endogenous fibroin L-chain, FibL-anti-WASP-scFv-Myc, and FibL-EGFP-His in each silk powder were confirmed by Western blotting with anti-FibL pAb (E). Immunoblots are representative of three independent experiments.

To examine whether S01 silk powder could specifically capture native WASP from mammalian immune cell extracts, RAW 264.7 cell lysate was immunoprecipitated with silk powder particles from W1, S01, and K27 strains or protein G-sepharose coupled with anti-WASP mAb or control mouse IgG. The immunocomplexes were pulled down and analyzed by Western blotting with anti-WASP pAb. In this assay, S01 silk powder particle immunoprecipitated native WASP as efficiently as anti-WASP-mAb–coupled protein G-sepharose (Figure 3, A and B). In addition, WASP-interacting protein (WIP), which is well known as a binding partner of WASP, was equivalently co-immunoprecipitated with WASP in the pull-down assay with S01 silk powder particle, as demonstrated in the assay using anti-WASP-mAb–coupled protein G-sepharose (Figure 3, C and D). These results suggested that S01 silk powder particle can efficiently capture the native WASP-WIP complex in mouse macrophage extract. A similar level of endogenous fibroin L-chain was detected by immunoblotting with anti-FibL pAb, indicating that an equal amount of silk powder was used in this assay (Figure 3E).

Given these observations, silk powder made from cocoons expressing scFv fused to fibroin protein may be potentially useful for developing an alternative reagent that retains affinity to target proteins comparable to that of conventional immunoprecipitation reagents, but costs less to manufacture using the described transgenic silkworm technology.

## Discussion

In the present study, we generated transgenic silkworm strain S01, which spun silk fibers containing anti-WASP-scFv–conjugated fibroin L-chain. The S01 silk powder not only specifically bound to the purified recombinant GST-WASP15 protein, but also efficiently immunoprecipitated native WASP-WIP complex in mouse RAW264.7 macrophage extract. These observations strongly suggest that scFv-conjugated silk fibroin proteins may be useful as a unique alternative affinity reagent.

To prepare silk powder, whole silk cocoons were dissolved in LiBr solution, dialyzed, freeze-dried, and mechanically crushed. Therefore, the resultant silk powder from each strain has similar composition and purity of both fibroin and sericin proteins. Although we do not know precisely how the fibroin and sericin proteins are combined and reconstituted during the processing of silk powder, at least some of the anti-WASP-scFv domains conjugated to the C-terminus of fibroin L-chains are exposed on the fracture surface of each silk powder particle. In addition, the anti-WASP-scFv domain spontaneously gains its proper conformation even in the conjugated form with fibroin L-chain, when silk proteins are expressed and secreted into the lumen of silk gland. Although some of them may be denatured, when silk proteins are dissolved in LiBr solution, most of scFv domains retain proper folding, which enables specific binding to the WASP molecule. As we demonstrated in the mammalian cells [20], the anti-WASP-scFv construct that we used to generate the affinity silk powder can regain its folding conformation, and binds to its target molecule in the cytoplasm, which is characterized by a very strong reducing condition. Therefore, an important factor for successful production of affinity silk powder is the capability of the employed scFv to fold autonomously to the correct conformation; this factor should be carefully considered when scFv conjugation is applied to other target molecules.

Semi-quantitative Western blot analysis demonstrated that S01 silk powders contain approximately 25% scFv-conjugated fibroin L-chains; the remaining 75% are non-transgenic endogenous fibroin L-chains. Although scFv-conjugated fibroin L-chains comprise a minority of the L-chains, sufficient and specific binding of each transgenic silk powder was demonstrated in the assay of both bacterial recombinant and mammalian native WASPs. In the latter target, the affinity silk powder particle also co-precipitated WIP, suggesting that these affinity silk powder particles may be useful in cellular and molecular immunology. Similarly, the scFv construct can also be conjugated to the fibroin H-chain (Sato et al. manuscript in preparation). The intercrossing of these transgenic silkworm strains would further increase the avidity of the affinity silk powders.

The traditional strategy used to generate affinity carriers using the antibody starts with the production of whole antibodies or antibody fragments in mammalian cells or bacteria, which is then followed by the isolation, purification, and surface immobilization of carriers by chemical coupling reactions. In sharp contrast to these complicated processes, affinity silk powders are ready-made reagents produced by transgenic silkworm technology, and require only a few purification steps. Therefore, affinity silk powders can be manufactured at a lower cost than those associated with traditional affinity carriers. After dissolving into solution, silk proteins can be fabricated into various material formats, including fibers, clothings, gels, sponges, and thin films [1]. For example, fibers, clothings or sponges produced from the scFv-conjugated affinity silk proteins can be used to capture and trap the antigens of pathogenic organisms, such as enterohemorrhagic bacteria (*E. coli*) or influenza virus. Fabrication into thin films can be applied to the construction of alternative detection systems for specific antigens associated with infectious disease, replacing classical enzyme-linked immunosorbent assay systems. Furthermore, recent advances in silk fibroin-derived nanoparticle technology [24], [25] may open a novel avenue to biomedical applications for scFv-conjugated affinity silk. Silk fibroin-derived nanoparticles have been used successfully as a drug-delivery platform [24], [25], and scFv-conjugated silk fibroin proteins would enhance the therapeutic effect by targeting specific cancer cells or neurodegenerative diseases, such as Alzheimer's and prion diseases. Further improvement of affinity silk technologies would provide novel materials that are highly biocompatible (as well as biodegradable) for the development of an affinity purification system, the diagnosis of diseases, the detection of pathogenic microorganisms, and the development of therapeutic strategies.

## Materials and Methods

### Plasmid construction

The primer sequences and procedure for construction of plasmids pBac[3xP3-DsRed2afm]-LLL-anti-WASP-scFv-Myc and pBac[3xP3-DsRed2afm]-LLL-EGFP-His is described in Supporting information Table S1.

### Generation of transgenic silkworms

Transgenic silkworms were generated as described elsewhere [26], with minor modifications. The transgene plasmid DNA and a helper plasmid vector pHA3PIG coding for *piggyBac* transposase [26], each dissolved in 5 mM KCl and 0.5 mM phosphate buffer (pH 7.0) at a concentration of 0.2 µg/µl, were mixed and injected into the fertilized eggs of the w1-pnd silkworm at 4 to 10 h post-oviposition. Hatched larvae (G0) were reared on an artificial diet (Nihon Nosan, Kanagawa, Japan) at 25°C until they developed into moths, and permitted to mate with each other. Using fluorescent microscopy (MZ16FA, Leica Microsystems, Wetzlar, Germany), G1 embryos were screened for transgenic individuals with DsRed2 expression 6 to 7 days after oviposition. Transgenic silkworms were reared and sib-mated for at least three generations. The experimental strain S01 carries the transgene coding for the fibroin L-chain fused with anti-WASP scFv-Myc (FibL-anti-WASP-scFv-Myc). The control strain K27 carries the transgene coding for the fibroin L-chain fused with EGFP-His (FibL-EGFP-His).

### Silk powder preparation

Three grams of silk cocoons obtained from wild-type w1-pnd (W1) and transgenic S01 and K27 silkworms were chopped into 2–3 mm squares and suspended in 50 ml of 9 M LiBr at 37°C on a rotator (10 rpm, 4 h) until dissolved. Resultant silk solutions were placed in a bag of cellulose dialysis membrane (Spectra/Por 1, MWCO = 6–8000; Spectrum Laboratories Inc., Rancho Dominguez, CA, USA), and dialyzed against 8 L of deionized water for 3 days at room temperature (RT) (the water was changed every 12 h). The dialyzed silk solution was air-dried at RT until it became gelatinous. Gelatinized silk was frozen at −80°C, dried using Freeze Dryer FDU-1100 (Tokyo Rikakikai, Tokyo, Japan), and crushed into powder using Wonder-Blender mixer WB-1 (Osaka Chemical, Osaka, Japan). The silk powders were directly observed using low-vacuum scanning electron microscopy (Miniscope TM-1000, Hitachi High-Technologies, Tokyo, Japan).

### Western blot analysis

Silk powder from W1, S01, and K27 strains was treated with 2× SDS sample buffer, separated by SDS-PAGE (12% gel), and transferred to a polyvinylidene difluoride (PVDF) membrane (Bio-Rad, Hercules, CA, USA). The blots were blocked with Blocking One (Nacalai Tesque, Kyoto, Japan) for 1 h at RT and then incubated with anti-fibroin L-chain (FibL) pAb (raised against a synthetic peptide representing fibroin L-chain residue 67–80), anti-Myc pAb, or anti-His pAb (MBL, Nagoya, Japan), followed by HRP-conjugated anti-rabbit Igs (Dako, Glostrup, Denmark). Immunoreactive proteins were detected using ECL reagent (GE Healthcare, Buckinghamshire, England).

### Construction of GST fusion proteins

cDNA fragments for mouse WASP exons 1–5 (aa 1–171, designated WASP15) and exons 6–9 (aa 172–313, designated WASP69) were generated by PCR from mRNA of C57BL/6 mouse spleen and cloned into the pGEX-4T-2 expression vector (GE Healthcare) (Table S1). The GST-WASP15 and GST-WASP69 fusion proteins were produced in BL21 *E. coli* cells and purified by glutathione-sepharose 4B affinity chromatography according to the manufacturer's instructions (GE Healthcare).

### GST fusion protein binding assay

After rinsing with 20% ethanol, 10 µg of each silk powder from W1, S01, and K27 strains was blocked with 1 ml of Blocking One in a 1.5 ml tube at RT on a rotator (10 rpm, 60 min). After centrifugation at 1,000 g for 3 min to remove blocking solution, each silk powder was incubated with 20 µg of GST, GST-WASP15, or GST-WASP69 fusion protein in 1 ml of blocking solution at RT on a rotator (10 rpm, 60 min). After five washes with TBST buffer (10 mM Tris-Hcl, pH8.0; 0.15 M NaCl; 0.05% Tween-20), silk powder was lysed with SDS sample buffer and immunoblotted with anti-GST, anti-Myc, or anti-His antibodies (MBL).

GST, GST-WASP15, and GST-WASP69 fusion proteins were incubated with 5 µg of anti-WASP mAb (a parental antibody for anti-WASP-scFv [20]) or control mouse IgG (Sigma-Aldrich, St Louis, MO, USA) at RT on a rotator (10 rpm, 60 min), and immunoprecipitated with 40 µl of protein G-sepharose (GE Healthcare). After five PBS washes, immunocomplexes were resuspended in SDS sample buffer and immunoblotted with ant-GST Ab.

### Immunoprecipitation

A murine macrophage RAW264.7 cell line [27], [28], which was obtained from American Type Culture Collection (ATCC No. TIB-71), was lysed with RIPA buffer [50 mM Tris-HCl, pH7.6; 150 mM NaCl; 1% Nonidet P-40; 0.5% sodium deoxycholate; and protease inhibitor cocktail (Nacalai Tesque)] on ice for 60 min. Cell lysates were centrifuged at 10,000 g for 10 min at 4°C, and the supernatants were incubated with W1, S01, or K27 silk powder pre-treated with blocking solution at RT on a rotator (10 rpm, 60 min). After five washes with TBST buffer, each silk powder was lysed with SDS sample buffer and immunoblotted with anti-WASP pAb (Upstate, Lake Placid, NY, USA), anti-WASP-interacting protein (WIP) pAb (Santa Cruz Biotechnology), or anti-fibroin-L-chain (FibL) pAb, and detected as described earlier.

## Supporting Information

Table S1 **In all primers, lower-case letters indicate restriction sites for** ***Eco*** **RI (gaattc),** ***Bam*** **HI (ggatcc),** ***Hind*** **III (aagctt),** ***Bgl*** **II (agatct), and** ***Not*** **I (gcggccgc).** cDNA fragments for the fibroin L-chain promoter region through the fibroin L-chain coding region (FibLpro–FibL) and the fibroin L-chain 3′-untranslated region (FibL-3′-UTR) were generated by PCR from pBac (3xP3-DsRed2+L-chain-GFP) [13] with the following primer sets (FibLpro–FibL: sense primer #1 and reverse primer #2, FibL-3′-UTR: sense primer #3 and reverse primer #4). A cDNA fragment for anti-WASP-scFv-Myc was generated by PCR from pCAG/anti-WASP-21HL [20] using sense primer #5 and reverse primer #6. These PCR products were digested with *Eco*RI-*Bam*HI, *Hind*III-*Bgl*II, and *Bam*HI-*Hind*III, respectively, and cloned together into the *Eco*RI-*Bgl*II site of the pBac[3xP3-DsRed2afm] vector. This construct was designated pBac[3xP3-DsRed2afm]-LLL-anti-WASP-scFv-Myc. The control plasmid vector, pBac[3xP3-DsRed2afm]-LLL-EGFP-His, was modified by the insertion of a 6×His tag sequence at the C-terminal coding region of fibroin L-chain and EGFP fusion protein in the original plasmid construct [13]. cDNA fragments for mouse WASP exons 1–5 (aa 1–171, designated WASP15) and exons 6–9 (aa 172–313, designated WASP69) were generated by PCR from mRNA of C57BL/6 mouse spleen with the following primers: WASP15, sense primer #7 and reverse primer #8; WASP69, sense primer #9 and reverse primer #10. These PCR products were digested with *Not*I, and cloned into the pGEX-4T-2 expression vector.
(DOC)
