# Supplementary material for: Production of scFv-Conjugated Affinity Silk Powder by Transgenic Silkworm Technology
Source: PLoS One. 2012 Apr 4;7(4):e34632. doi: 10.1371/journal.pone.0034632 (PMC3319607; doi:10.1371/journal.pone.0034632)
Supplement: Table S1 — In all primers, lower-case letters indicate restriction sites for Eco RI (gaattc), Bam HI (ggatcc), Hind III (aagctt), Bgl II (agatct), and Not I (gcggccgc). cDNA fragments for the fibroin L-chain promoter region through the fibroin L-chain coding region (FibLpro–FibL) and the fibroin L-chain 3′-untranslated region (FibL-3′-UTR) were generated by PCR from pBac (3xP3-DsRed2+L-chain-GFP) [13] with the following primer sets (FibLpro–FibL: sense primer #1 and reverse primer #2, FibL-3′-UTR: sense primer #3 and reverse primer #4). A cDNA fragment for anti-WASP-scFv-Myc was generated by PCR from pCAG/anti-WASP-21HL [20] using sense primer #5 and reverse primer #6. These PCR products were digested with EcoRI-BamHI, HindIII-BglII, and BamHI-HindIII, respectively, and cloned together into the EcoRI-BglII site of the pBac[3xP3-DsRed2afm] vector. This construct was designated pBac[3xP3-DsRed2afm]-LLL-anti-WASP-scFv-Myc. The control plasmid vector, pBac[3xP3-DsRed2afm]-LLL-EGFP-His, was modified by the insertion of a 6×His tag sequence at the C-terminal coding region of fibroin L-chain and EGFP fusion protein in the original plasmid construct [13]. cDNA fragments for mouse WASP exons 1–5 (aa 1–171, designated WASP15) and exons 6–9 (aa 172–313, designated WASP69) were generated by PCR from mRNA of C57BL/6 mouse spleen with the following primers: WASP15, sense primer #7 and reverse primer #8; WASP69, sense primer #9 and reverse primer #10. These PCR products were digested with NotI, and cloned into the pGEX-4T-2 expression vector. (DOC) [file pone.0034632.s001.doc]

Table S1. Specific oligonucleotide primers used to amplify fibroin L-chain fragments, anti-WASP-scFv-Myc, WASP15, and WASP69

| Primer 1 5’-CTGCgaattcGGCTCATATGAGATCTGG-3’ |
| --- |
| Primer 2 5’-CCGGggatccGCGTCATTACCGTTGCC-3’ |
| Primer 3 5’-GATCaagcttATCGATACCGTCGAGATCC-3’ |
| Primer 4 5’-GTCGagatctCATGACAACAGTACCG-3’ |
| Primer 5 5’-CTCCggatccCGCCACCATGGAGGTTCAGCTGCAG-3’ |
| Primer 6 5’-CACCCaagcttCTACAGGTCCTCCTCGCTGATCAG-3’ |
| Primer 7 5’-CGAATgcggccgcAATGAATAGTGGCCCTG-3’ |
| Primer 8 5’-CGAATgcggccgcTCACTCCTCATTGATTGG-3’ |
| Primer 9 5’-CGAATgcggccgcAATGAGAAGAGGAGGGCTCCC-3’ |
| Primer 10 5’-CGAATgcggccgcTCACTCTTGGCGCCTCATCTC-3’ |
